# Supplementary material for: Force-modulated structural landscape of the catch bonding F-actin crosslinker α-actinin-4
Source: bioRxiv. 2026 Apr 12:2026.03.04.709699. Preprint. [Version 3] doi: 10.64898/2026.03.04.709699 (PMC13001351; doi:10.64898/2026.03.04.709699)
Supplement: Supplement 4 [file NIHPP2026.03.04.709699v3-supplement-4.pdf]

bioRxiv preprint doi: https://doi.org/10.64898/2026.03.04.709699; this version posted April 12, 2026. The copyright holder for this preprint (which was not certified by peer review) is the author/funder, who has granted bioRxiv a license to display the preprint in perpetuity. It is made available under aCC-BY 4.0 International license.

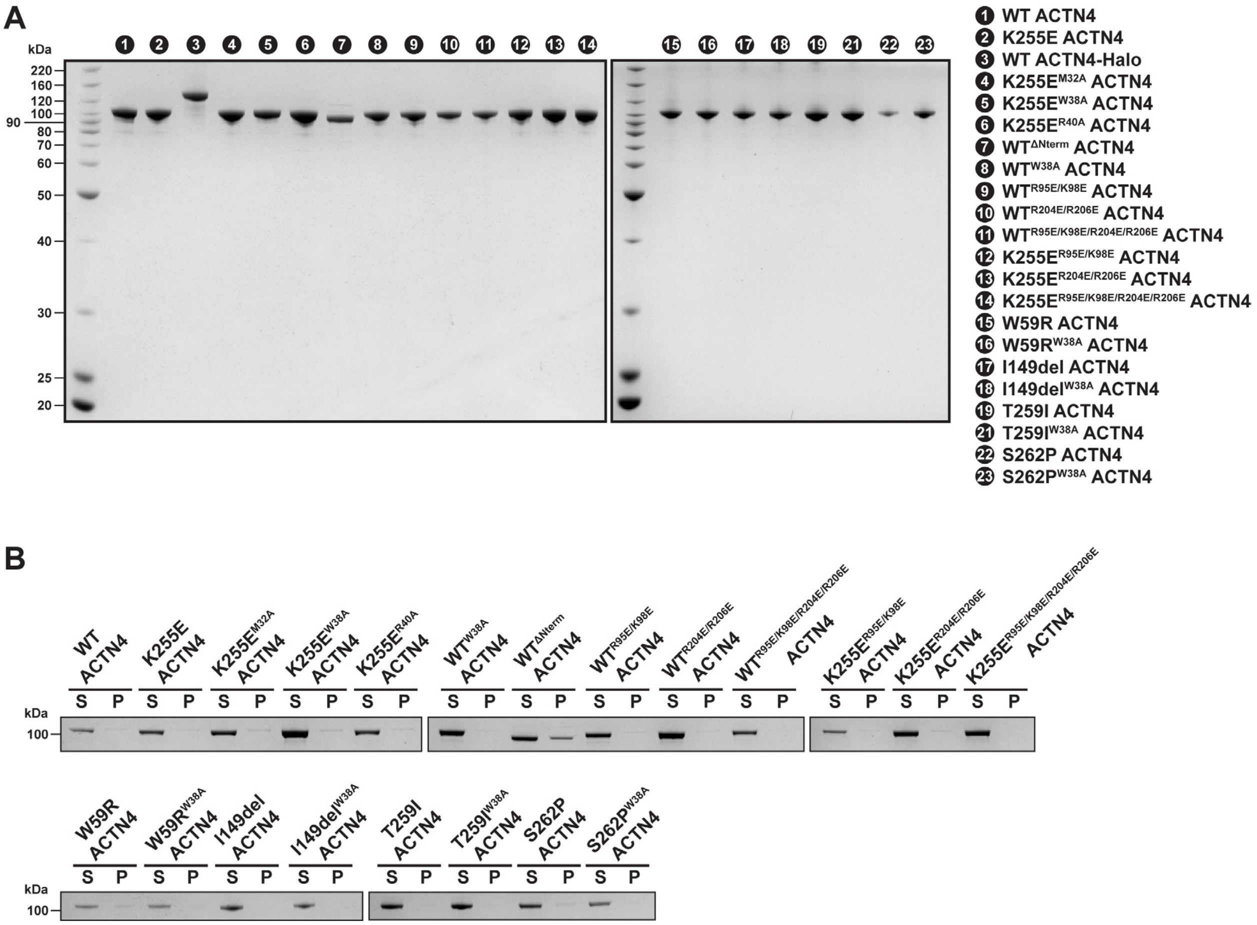

Figure S1: ACTN4 purification and sedimentation without actin. (A) SDS-PAGE of all purified ACTN4 proteins used in this study. **(B)** SDS-PAGE of control sedimentation assays performed in the absence of F-actin. WT$^{\Delta N\text{-}term}$ ACTN4 was the only construct which displayed substantial pelleting.

bioRxiv preprint doi: https://doi.org/10.64898/2026.03.04.709699; this version posted April 12, 2026. The copyright holder for this preprint (which was not certified by peer review) is the author/funder, who has granted bioRxiv a license to display the preprint in perpetuity. It is made available under aCC-BY 4.0 International license.

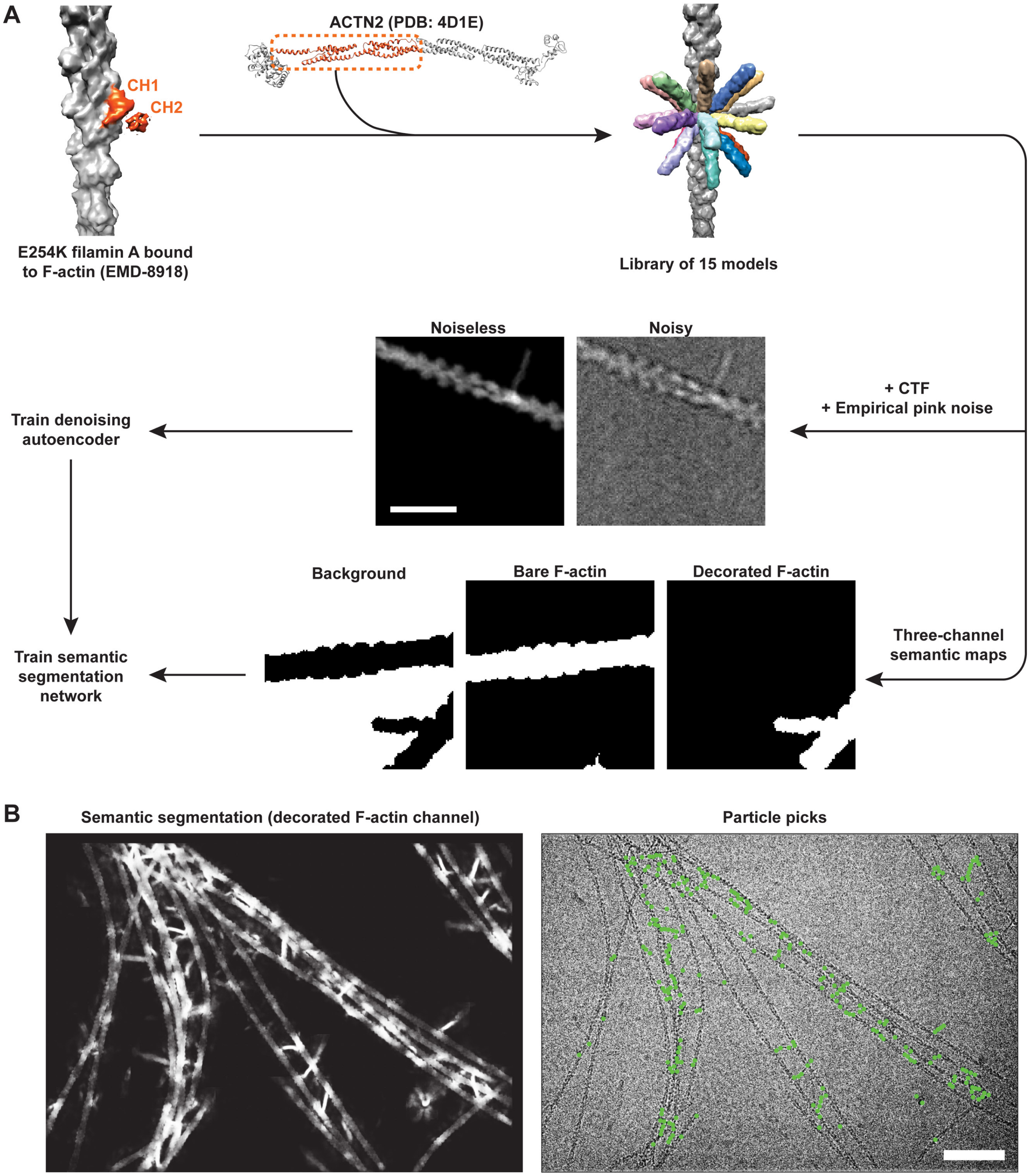

Figure S2: Neural network-based particle picker and example performance on experimental cryo-EM data. (A) Schematic of synthetic data generation and neural network training. Scale bar, 20 nm. **(B)** Network performance on a representative micrograph from the K255E ACTN4–F-actin dataset. Scale bar, 80 nm.

bioRxiv preprint doi: https://doi.org/10.64898/2026.03.04.709699; this version posted April 12, 2026. The copyright holder for this preprint (which was not certified by peer review) is the author/funder, who has granted bioRxiv a license to display the preprint in perpetuity. It is made available under aCC-BY 4.0 International license.

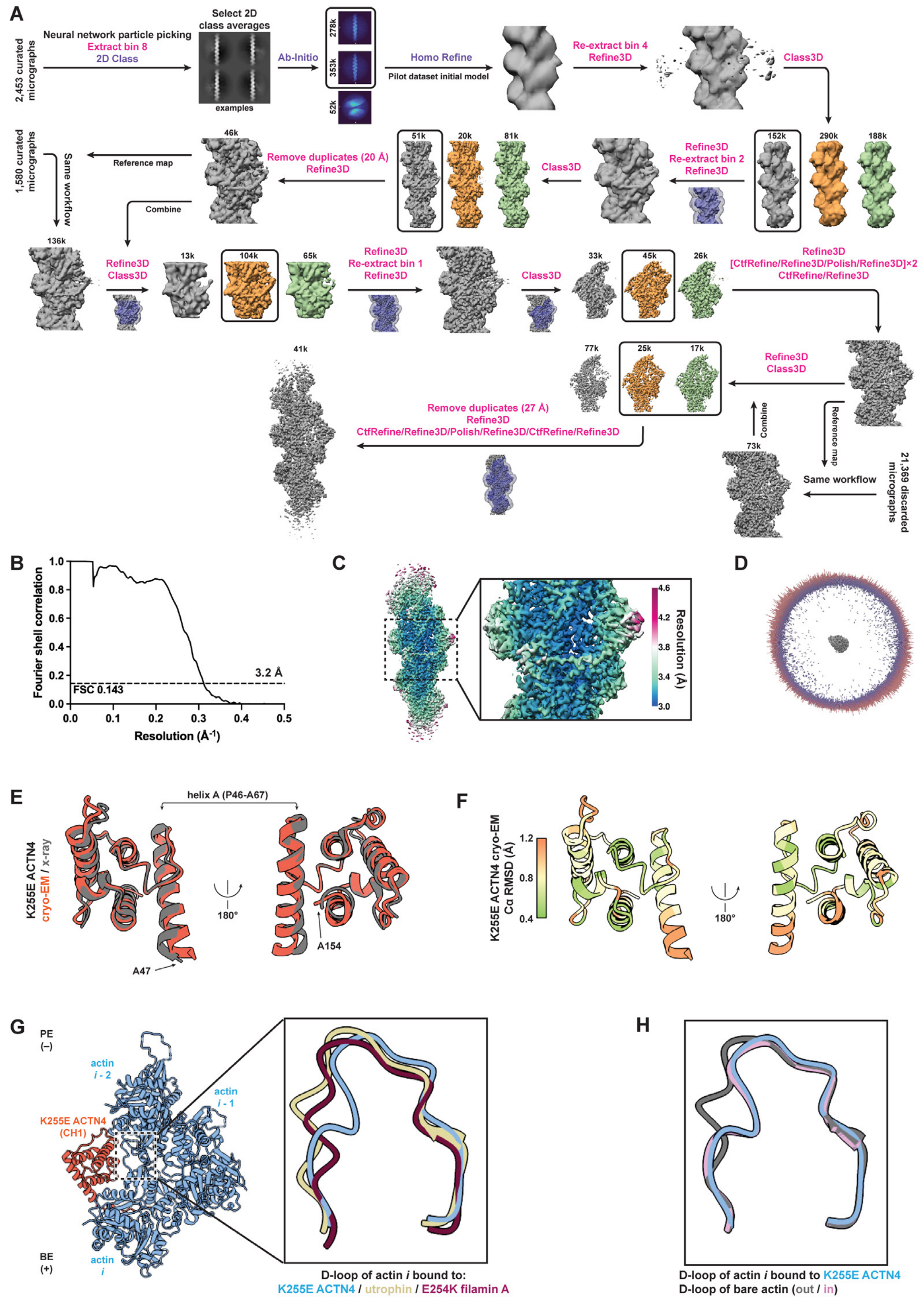

Figure S3: Cryo-EM data processing and analysis of K255E ACTN4–F-actin. (A) Data processing workflow. Each mask is displayed at the first step where it was used, superimposed on the corresponding reference. Jobs performed in RELION and cryoSPARC are indicated in magenta and purple text, respectively. **(B)** Fourier shell correlation (FSC) curve. **(C)** Local resolution assessment. **(D)** 3D angular distribution. **(E)** Superposition of K255E ACTN4 CH1 from the unbound ABD crystal structure[34] (PDB 2R0O) and F-actin bound cryo-EM reconstruction (this study). **(F)** C_α root mean square deviation (RMSD) between superimposed structures from **E**.

bioRxiv preprint doi: https://doi.org/10.64898/2026.03.04.709699; this version posted April 12, 2026. The copyright holder for this preprint (which was not certified by peer review) is the author/funder, who has granted bioRxiv a license to display the preprint in perpetuity. It is made available under aCC-BY 4.0 International license.

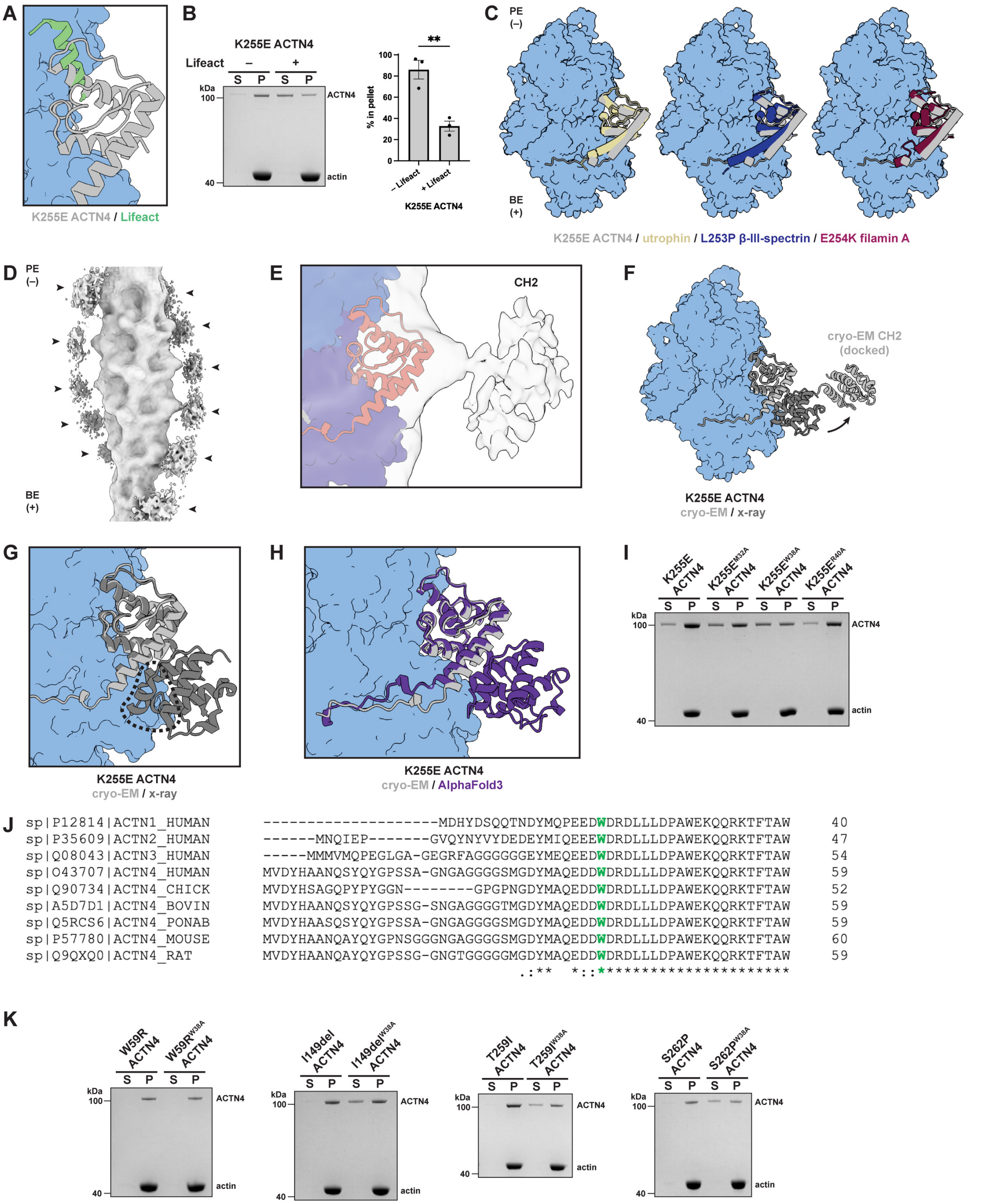

Figure S4: Additional analysis of the K255E ACTN4–F-actin structure. (A) Superposition of the Lifeact–F-actin complex[42] (PDB 7AD9) and the K255E ACTN4–F-actin structure (this study) highlights their overlapping binding sites. **(B)** Representative SDS-PAGE and quantification of competition F-actin co-sedimentation assay between Lifeact and K255E ACTN4. S, supernatant. P, pellet. Upper band (105 kDa) is ACTN4, and lower band (42 kDa) is actin. Data are presented as mean ± SEM (n = 3). Conditions were compared by unpaired t test, $^{**}P < 0.01$. **(C)** F-actin-bound

bioRxiv preprint doi: https://doi.org/10.64898/2026.03.04.709699; this version posted April 12, 2026. The copyright holder for this preprint (which was not certified by peer review) is the author/funder, who has granted bioRxiv a license to display the preprint in perpetuity. It is made available under aCC-BY 4.0 International license.

utrophin[39] (PDB 6M5G), L253P β-III-spectrin[37] (PDB 6ANU), and E254K FLNA[36] (PDB 6D8C) superimposed on F-actin-bound K255E ACTN4 (aligned on actin). Actins from the K255E ACTN4–F-actin structure are displayed. BE (+), barbed (plus) end; PE (−), pointed (minus) end. **(D)** Cryo-EM map derived from pilot K255E ACTN4–F-actin dataset, which featured excess K255E ACTN4 decoration. Diffuse densities consistent with displaced CH2 are highlighted (arrowheads). **(E)** K255E ACTN4–F-actin atomic model docked into pilot cryo-EM map from **D**, with unoccupied density attributable to CH2. **(F)** Approximate docking of CH2 into cryo-EM density alongside the K255E ACTN4–F-actin atomic model (light grey) highlights ABD opening relative to the unbound closed conformation[34] (PDB 2R0O, superimposed on CH1). **(G)** Superposition of the unbound K255E ACTN4 ABD on F-actin-bound K255E ACTN4 (aligned on CH1) highlights region with clashes between CH2 and F-actin in the closed conformation. **(H)** AlphaFold3 predicted structure of the K255E ACTN4 ABD (residues 1-269) bound to F-actin (five human α-actin-1 subunits) superimposed the cryo-EM structure (aligned on actin). CH2 domain of the AlphaFold3 predicted structure remains in a closed conformation. The AlphaFold3-predicted NTE binding mode also differs from that in our cryo-EM structure. Residues 1-30 of K255E ACTN4 were disordered and extended away from F-actin in the predicted structure, and they are hidden for clarity. **(I)** SDS-PAGE of representative F-actin co-sedimentation assay for alanine mutations in the K255E ACTN4 NTE. Related to Fig. 1H. **(J)** Sequence alignment of NTEs of ACTN4 proteins from indicated species and human ACTN1-3. W38 (human ACTN4 numbering) is conserved across ACTNs (green). (**K**) SDS-PAGE of representative F-actin co-sedimentation assay for indicated FSGS-causing ACTN4 mutations and their corresponding W38A variants. Related to Fig. 1J.

bioRxiv preprint doi: https://doi.org/10.64898/2026.03.04.709699; this version posted April 12, 2026. The copyright holder for this preprint (which was not certified by peer review) is the author/funder, who has granted bioRxiv a license to display the preprint in perpetuity. It is made available under aCC-BY 4.0 International license.

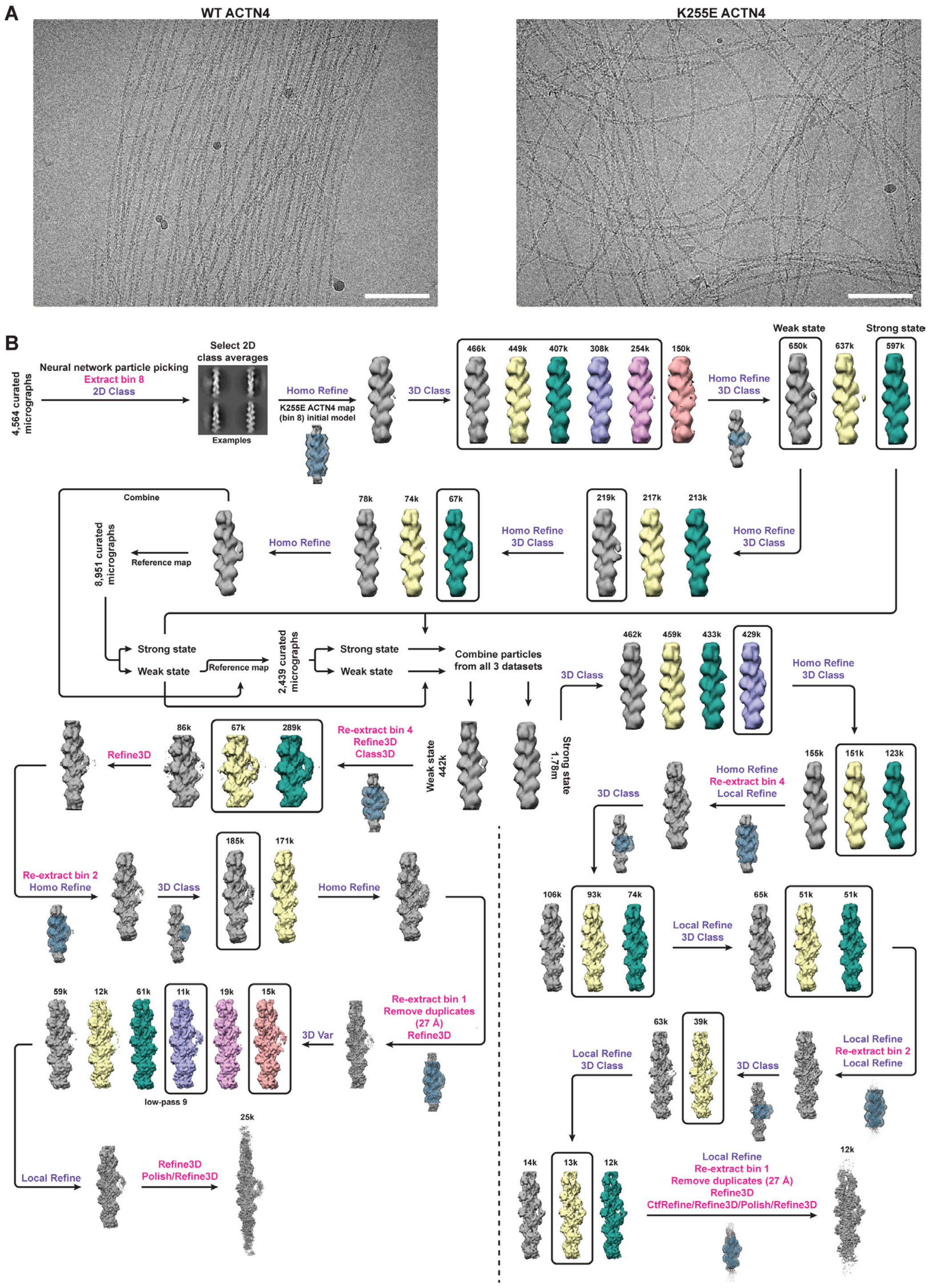

Figure S5: Cryo-EM and image processing for wild-type ACTN4–F-actin in the absence of myosin motors. (A) Representative micrographs from wild-type ACTN4–F-actin and K255E ACTN4–F-actin datasets, highlighting differential filament network organization. Scale bars, 100 nm. **(B)** Image processing workflow for recovering both weak and strong state cryo-EM maps. Each mask is displayed at the first step where it was used, superimposed on the corresponding reference. Jobs performed in RELION and cryoSPARC are indicated in magenta and purple text, respectively.

bioRxiv preprint doi: https://doi.org/10.64898/2026.03.04.709699; this version posted April 12, 2026. The copyright holder for this preprint (which was not certified by peer review) is the author/funder, who has granted bioRxiv a license to display the preprint in perpetuity. It is made available under aCC-BY 4.0 International license.

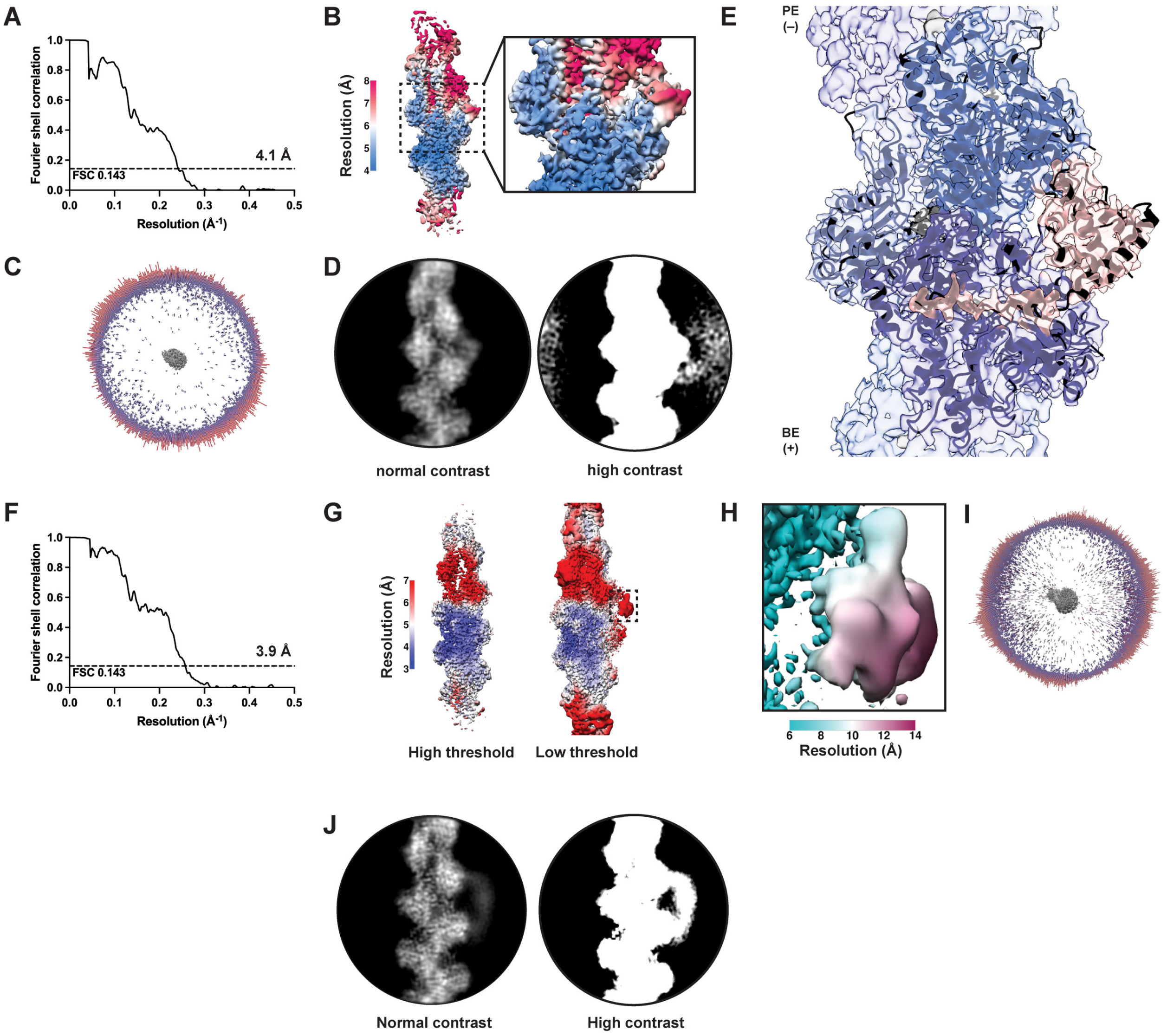

Figure S6: Cryo-EM data analysis for wild-type ACTN4–F-actin in the absence of myosin motors. (A) FSC curve for strong state map. **(B)** Local resolution assessment for strong state map. **(C)** 3D angular distribution of particles contributing to the strong state map. **(D)** 2D class average of particles contributing to the strong state map, viewed at different thresholds. At low threshold, diffuse density is visible that likely corresponds to CH2 (similar as visible for K255E ACTN4, Fig. 1D). **(E)** Atomic model of K255E ACTN4 bound to F-actin docked into the wild-type ACTN4 strong state map. BE (+), barbed (plus) end; PE (–), pointed (minus) end. **(F)** FSC curve for weak state map. **(G)** Overall local resolution assessment for weak state map. Note that color scale does not capture resolution range of boxed ABD region. **(H)** Local resolution assessment for ABD density in box from **G**, with adjusted color scale to capture resolution gradient in this region. **(I)** 3D angular distribution of weak state map. **(J)** 2D class average of particles contributing to the weak state map, viewed at different thresholds. Diffuse density distal from the filament surface is not present at low threshold (as is observed in the strong state), consistent with CH2 engaging F-actin in the weak state.

bioRxiv preprint doi: https://doi.org/10.64898/2026.03.04.709699; this version posted April 12, 2026. The copyright holder for this preprint (which was not certified by peer review) is the author/funder, who has granted bioRxiv a license to display the preprint in perpetuity. It is made available under aCC-BY 4.0 International license.

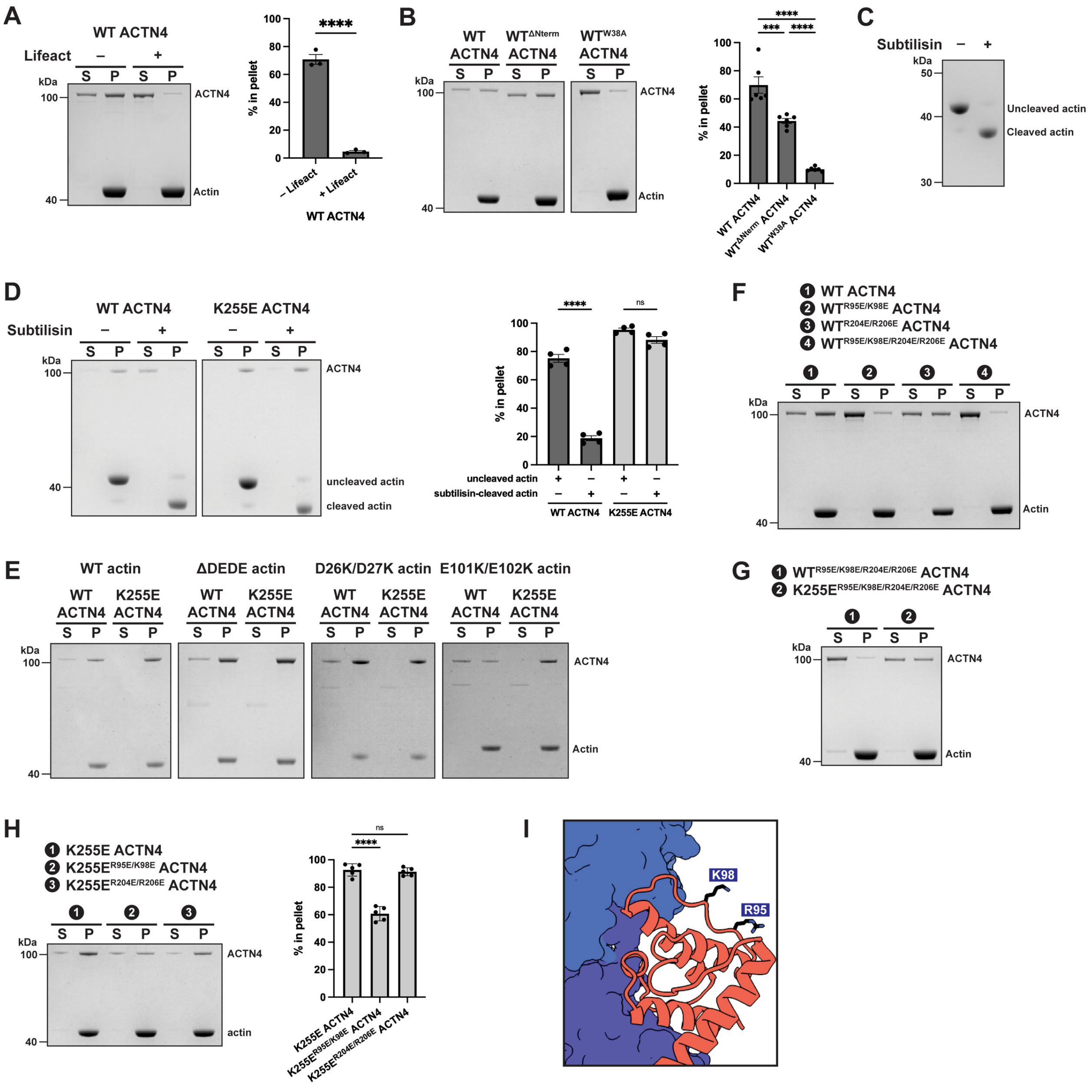

Figure S7: Biochemical assays to dissect F-actin binding modes of wild-type ACTN4. (A) SDS-PAGE of representative co-sedimentation assay and quantification of Lifeact competitive F-actin binding with wild-type ACTN4. S, supernatant. P, pellet. Upper band (105 kDa) is ACTN4, and lower band (42 kDa) is actin. Conditions were compared by unpaired t test (n = 3). **(B)** SDS-PAGE of representative co-sedimentation assay and quantification of F-actin binding by NTE point mutations and truncation in the context of wild-type ACTN4. Conditions were compared by ordinary one-way ANOVA with Tukey's correction (n = 6). **(C)** SDS-PAGE of uncleaved and subtilisin-cleaved actin. **(D)** SDS-PAGE of representative co-sedimentation assay and quantification of uncleaved versus subtilisin-cleaved F-actin binding by wild-type and K255E ACTN4. Conditions were compared by ordinary two-way ANOVA with Šídák's correction (n = 4). **(E)** Representative SDS-PAGE of wild-type and K255E ACTN4 co-sedimentation assay with F-actin featuring charge reversal mutations (in recombinant human α-actin-1). Related to Fig. 2D. **(F)** Representative SDS-

bioRxiv preprint doi: https://doi.org/10.64898/2026.03.04.709699; this version posted April 12, 2026. The copyright holder for this preprint (which was not certified by peer review) is the author/funder, who has granted bioRxiv a license to display the preprint in perpetuity. It is made available under aCC-BY 4.0 International license.

PAGE of F-actin co-sedimentation assay for ACTN4 charge reversal mutations in the context of the wild-type ACTN4 background. Related to Fig. 2G. **(G)** Representative SDS-PAGE of F-actin co-sedimentation assay for simultaneous charge reversal mutations in both CHDs of wild-type and K255E ACTN4. Related to Fig. 2H. **(H)** Representative SDS-PAGE of F-actin co-sedimentation assay and quantification for charge reversal mutations in K255E ACTN4. Conditions were compared by ordinary one-way ANOVA with Dunnett's correction (n = 5). **(I)** Residues contributing to the positive patch on CH1 are displayed on the K255E ACTN4–F-actin structure, highlighting how they are facing away from the F-actin surface in the strong state. For quantification of co-sedimentation assays throughout the figure, data are presented as mean ± SEM. ***$P < 0.001$, ****$P < 0.0001$, ns = not significant ($P \geq 0.05$)

bioRxiv preprint doi: https://doi.org/10.64898/2026.03.04.709699; this version posted April 12, 2026. The copyright holder for this preprint (which was not certified by peer review) is the author/funder, who has granted bioRxiv a license to display the preprint in perpetuity. It is made available under aCC-BY 4.0 International license.

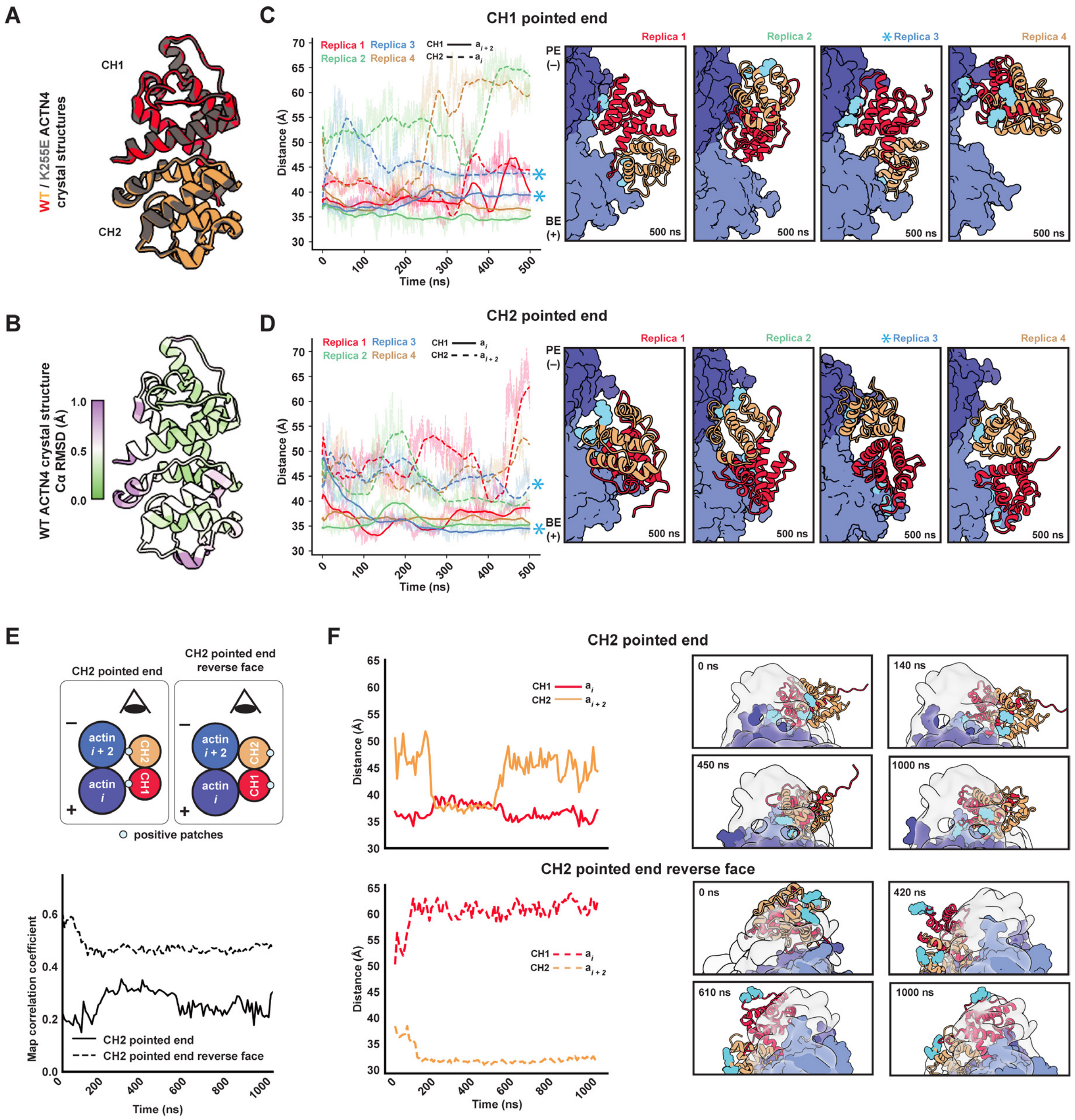

Figure S8: Additional MD analysis of the weak-binding state. (A) Superposition of wild-type[35] (PDB 6O31) and K255E[34] (PDB 2R0O) ACTN4 ABD crystal structures. **(B)** C_α RMSD between superimposed structures from **A**. **(C-D)** Plots of CH domain distance to the nearest actin across four replica simulations (left) and snapshots corresponding to the final frame of each 500 ns trajectory (right) for the CH1 pointed end **(C)** and CH2 pointed end **(D)** orientations. Replicas indicated with asterisks were run for an additional 1 µs. BE (+), barbed (plus) end; PE (−), pointed (minus) end. **(E)** Cartoon of additional ACTN4 ABD orientations examined in MD simulations of weak state (top). Map correlation coefficients from 1 µs MD simulations of ACTN4 ABD in orientations from (bottom). (**F**) Plots of CH domain distance to the nearest actin subunit (left) and representative simulation snapshots overlaid with the cryo-EM map (right) of the CH2 pointed end (top) and CH2 pointed end reverse face (bottom) orientations. Snapshots are from the view indicated in **E**. Distances were calculated between the centers of mass of C_α atoms. Cyan residues indicate positive amino acids highlighted in Fig. 2B.

bioRxiv preprint doi: https://doi.org/10.64898/2026.03.04.709699; this version posted April 12, 2026. The copyright holder for this preprint (which was not certified by peer review) is the author/funder, who has granted bioRxiv a license to display the preprint in perpetuity. It is made available under aCC-BY 4.0 International license.

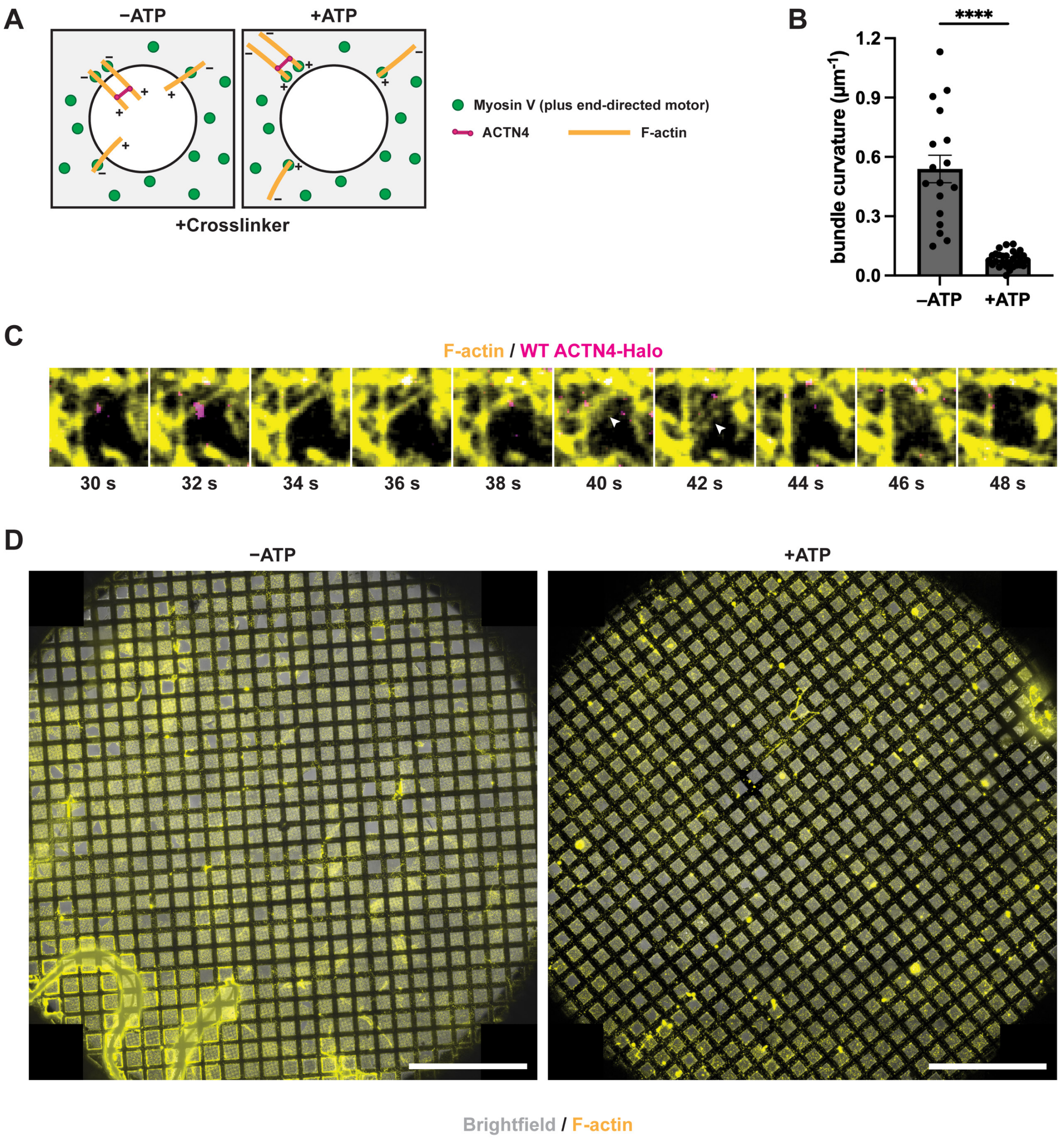

Figure S9: Force reconstitution assay on cryo-EM grids. (A) Schematic of force reconstitution assay behavior when crosslinkers form parallel F-actin bundles, which are anticipated to glide on to the carbon film in a manner similar to unbundled filaments. **(B)** Example of force-induced bundle ruptures (arrowheads), which occurred at later time points in the hole displayed in Fig. 3C (bottom). **(C)** Quantification of bundle curvature from epifluorescence of force reconstitution assays in the presence of wild-type ACTN4-Halo with or without ATP. Analysis was performed on 5 and 2 movies for −ATP and +ATP conditions, respectively. Data are presented as mean ± SEM (n = 17 and 28 for −ATP and +ATP conditions, respectively). Conditions were compared by unpaired t test, ****$P < 0.0001$. **(D)** Cryo-fluorescence images of −ATP and +ATP grids. Whole-grid atlases were generated by maximum intensity projection and stitching of images of smaller regions. Brightfield and F-actin (Alexa Fluor Plus 555 phalloidin) channels are displayed. Scale bars, 500 µm.

bioRxiv preprint doi: https://doi.org/10.64898/2026.03.04.709699; this version posted April 12, 2026. The copyright holder for this preprint (which was not certified by peer review) is the author/funder, who has granted bioRxiv a license to display the preprint in perpetuity. It is made available under aCC-BY 4.0 International license.

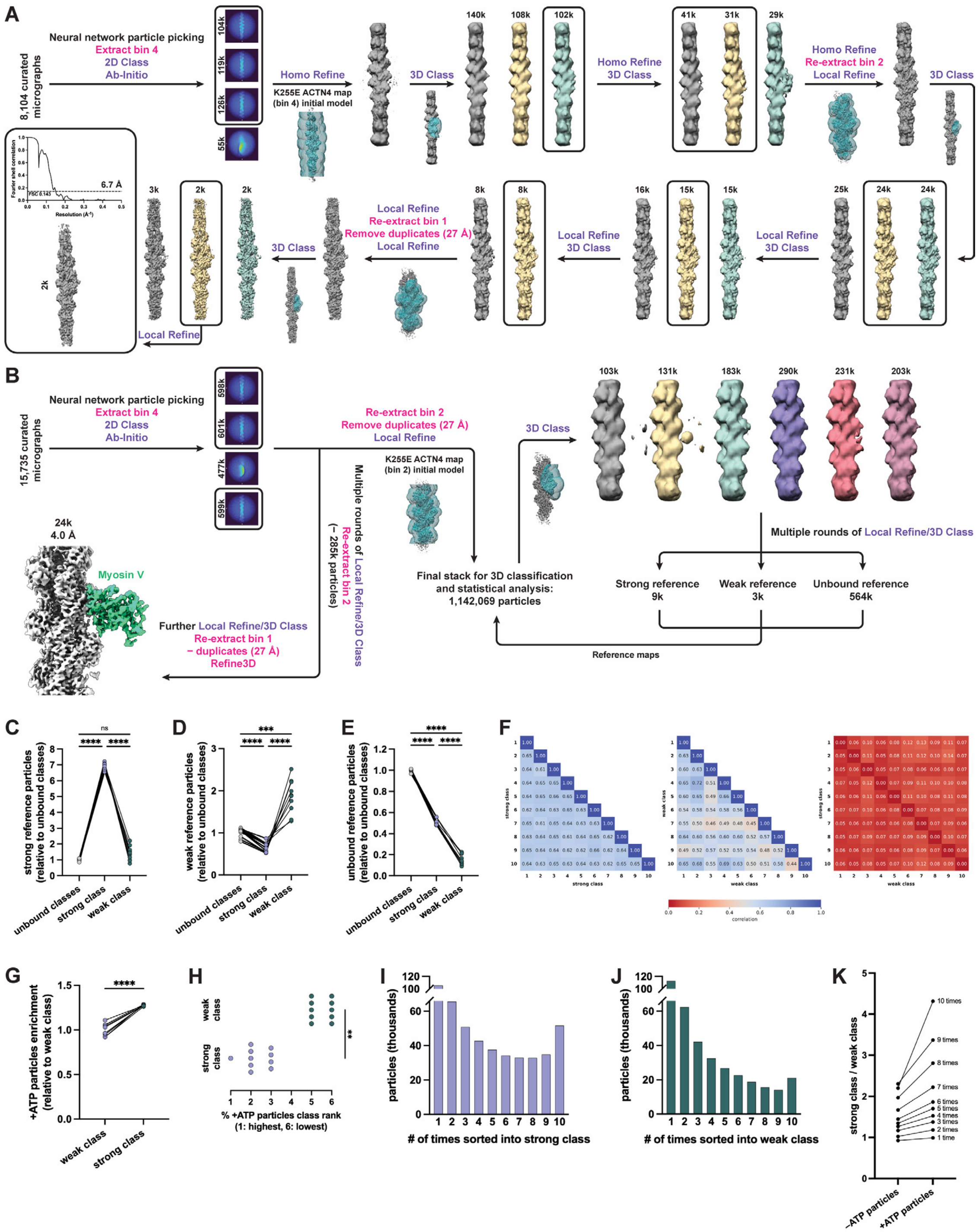

Figure S10: Cryo-EM data processing and analysis for wild-type ACTN4–F-actin in the presence of myosin motor forces. (A) Image processing workflow for analyzing wild-type ACTN4 bound to F-actin from the +ATP dataset alone. Each mask is shown at the first step it was used, superimposed on the corresponding reference. The FSC curve for the final reconstruction is also displayed. **(B)** Detailed workflow for processing combined −ATP and +ATP datasets in preparation for quantification by 3D classification, featuring data cleaning and generation of references for strong, weak, and unbound states. Jobs performed in RELION and cryoSPARC are displayed in magenta

bioRxiv preprint doi: https://doi.org/10.64898/2026.03.04.709699; this version posted April 12, 2026. The copyright holder for this preprint (which was not certified by peer review) is the author/funder, who has granted bioRxiv a license to display the preprint in perpetuity. It is made available under aCC-BY 4.0 International license.

and purple, respectively. **(C-E)** Post-supervised 3D classification assignments of particles originally associated with strong (**C**), weak (**D**), and unbound (**E**) references. Data are normalized to class sizes and expressed as fold-enrichment relative to unbound classes. Conditions were compared by repeated measures one-way ANOVA with Geisser-Greenhouse and Tukey's corrections. **(F)** Correlation matrices assessing similarity of class assignments between independent 3D classification runs. Analysis is based on number of shared particles, normalized to class size displayed on horizontal axis. **(G)** Quantification of +ATP dataset particle enrichment in weak and strong classes (expressed as fold change relative to weak class). Conditions were compared by ratio paired t test. **(H)** For each 3D classification run, individual classes were ranked by percent of particles derived from the +ATP dataset and plotted. Rankings for strong and weak classes within runs were then compared by Wilcoxon matched-pairs signed rank test. **(I-J)** Quantification of per-particle consistency of class assignment across classification runs for the strong **(I)** and weak **(J)** classes. **(K)** Analysis of the relationship between how consistently particles appear in their respective class (I and J) and the shift in class assignments between -ATP and +ATP datasets. A larger magnitude ATP induced shift towards the strong class is observed for more consistently assigned particles. Related to Fig. 3G. For all statistical comparisons throughout the figure, $n = 10$ independent 3D classification runs. ****$P < 0.0001$, ns = not significant ($P \geq 0.05$).

bioRxiv preprint doi: https://doi.org/10.64898/2026.03.04.709699; this version posted April 12, 2026. The copyright holder for this preprint (which was not certified by peer review) is the author/funder, who has granted bioRxiv a license to display the preprint in perpetuity. It is made available under aCC-BY 4.0 International license.

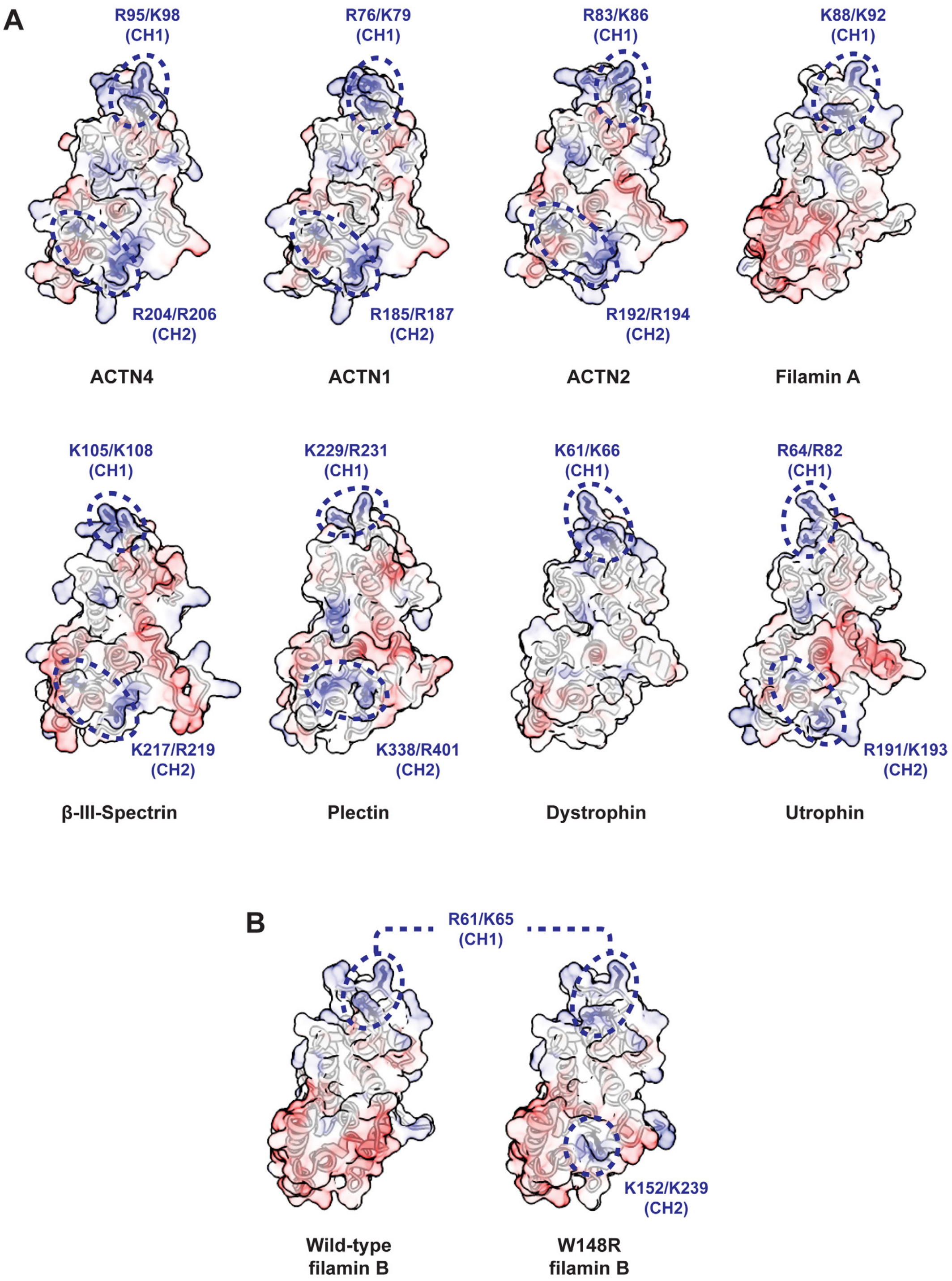

Figure S11: Charge distributions across tandem CHD ABDs. (A) Comparison of electrostatic potential surface maps of wild-type ACTN4[35] (PDB 6O31), ACTN1[32] (PDB 2EYI), ACTN2[90] (PDB 4D1E), filamin A[33] (PDB 3HOP), β-III-spectrin[91] (AlphaFold DB/Uniprot O15020), plectin[92] (PDB 1MB8), dystrophin[93] (PDB 9D58), and utrophin (AlphaFold3 prediction of residues 31-255) ABDs. **(B)** Comparison of electrostatic potential surface maps of wild-type (PDB 2WA5) and W148R (PDB 2WA6) filamin B ABDs[62].

bioRxiv preprint doi: https://doi.org/10.64898/2026.03.04.709699; this version posted April 12, 2026. The copyright holder for this preprint (which was not certified by peer review) is the author/funder, who has granted bioRxiv a license to display the preprint in perpetuity. It is made available under aCC-BY 4.0 International license.

Table S1: Cryo-EM data collection, refinement, and validation statistics.

	K255E ACTN4 bound to F-actin EMDB: XXXX PDB: XXXX	Wild-type ACTN4 bound to F-actin (strong state) EMDB: XXXX	Wild-type ACTN4 bound to F-actin (weak state) EMDB: XXXX	Wild-type ACTN4 bound to F-actin under force (+ATP dataset only) EMDB: XXXX
**Data collection and processing**				
Microscope	Titan Krios	Titan Krios	Titan Krios	Titan Krios
Detector	K3	K3	K3	K3
Magnification	81,000	81,000	81,000	81,000
Voltage (kV)	300	300	300	300
Electron exposure ($e^-/Å^2$)	49.238	49.238	49.238	49.238
Defocus range (μm)	−0.8 to −2.2	−0.8 to −2.2	−0.8 to −2.2	−0.8 to −2.2
Pixel size (Å)	1.09	1.09	1.09	1.09
Symmetry imposed	C1	C1	C1	C1
Micrographs (no.)	25,402	15,954	15,954	8,104
Initial particle images (no.)	7,291,435	9,389,999	9,389,999	1,734,836
Final particle images (no.)	41,450	25,362	12,309	2,078
Map resolution (Å)	3.2	4.1	3.9	6.7
FSC threshold	0.143	0.143	0.143	0.143
Map resolution range (Å)	3.0–5.8	4.2–27.1	3.5–18.0	4.2–16.2
**Refinement**				
Initial model used (PDB code)	8D14, 2R0O	-	-	-
Model resolution (Å)	3.2	-	-	-
FSC threshold	0.5	-	-	-
Map sharpening *B* factor ($Å^2$)	−41.7	−49.5	−50.6	-
Model composition	3 actin protomers, 1 K255E ACTN4	-	-	-
Non-hydrogen atoms	9855	-	-	-
Protein residues	1243	-	-	-
Ligands	3 Mg^{2+}, 3 ADP, 3 PO_4^{3-}1 phalloidin	-	-	-
B factors ($Å^2$)				
Protein	40.57	-	-	-
Ligand	27.24	-	-	-
RMSD				
Bond lengths (Å)	0.002	-	-	-
Bond angles (°)	0.545	-	-	-
**Validation**				
MolProbity score	1.21	-	-	-
Clashscore	3.78	-	-	-
Poor rotamers (%)	0.67	-	-	-
Ramachandran plot				
Favored (%)	97.79	-	-	-
Allowed (%)	2.21	-	-	-
Disallowed (%)	0.00	-	-	-
